# SWiss Atorvastatin and Interferon Beta-1b Trial In Multiple Sclerosis (SWABIMS) - rationale, design and methodology

**DOI:** 10.1186/1745-6215-10-115

**Published:** 2009-12-14

**Authors:** Christian P Kamm, Heinrich P Mattle, SWABIMS Study Group

**Affiliations:** 1 Department of Neurology, Inselspital, Bern University Hospital, and University of Bern, Switzerland

## Abstract

**Background:**

Statins have anti-inflammatory and immunomodulatory properties in addition to their lipid-lowering effects. Currently, the effects of statins on multiple sclerosis are still controversial. Therefore, randomized clinical trials are needed to provide better evidence on the therapeutic potential of statins in multiple sclerosis. The SWiss Atorvastatin and Interferon Beta-1b trial in Multiple Sclerosis (SWABIMS) evaluates the efficacy, safety and tolerability of atorvastatin 40 mg per os daily and subcutaneous interferon beta-1b every other day compared to monotherapy with subcutaneous interferon beta-1b every other day in patients with relapsing-remitting multiple sclerosis.

**Methods/Design:**

SWABIMS is a multi-centre, randomized, parallel-group, rater-blinded, Phase IIb-study conducted in eight hospitals in Switzerland. 80 treatment naïve patients with relapsing-remitting forms of multiple sclerosis will receive subcutaneous interferon beta-1b for three months. Afterwards, they are randomized into two equal-sized parallel arms, receiving atorvastatin 40 mg/d or not in addition to interferon beta-1b for another 12 months. Disease activity measured by the proportion of patients with new T2 lesions is the primary endpoint.

**Discussion:**

SWABIMS is designed to give further information about the therapeutic effect of atorvastatin 40 mg per os daily as add-on therapy to interferon beta-1b in patients with relapsing-remitting multiple sclerosis. Furthermore important safety and tolerability data will be generated.

**Trial Registration:**

http://www.clinicaltrials.gov. Identifier: NCT00942591; Swissmedic reference number: 2005DR2119

## Background

Multiple Sclerosis (MS) is a chronic inflammatory disorder of the central nervous system involving autoimmune mechanisms [1]. At present, there is no cure for multiple sclerosis and the management of MS patients requires treatment with disease-modifying agents such as interferon beta (IFNB), glatiramer acetate, natalizumab or immunsuppressants such as mitoxantrone, azathioprine or methotrexate.

Interferon beta-1b (Betaseron^®^, Betaferon^®^) is a non-glycosylated recombinant human IFNB approved for high-frequency subcutaneous (sc) administration to treat MS (250 micro g, 8 million International Units [MIU] every other day [e.o.d]). It reduces the relapse rate and increases the proportion of relapse-free patients with relapsing-remitting MS (RRMS) compared to placebo. It also reduces relapse severity, hospitalisations, and disease activity assessed by magnetic resonance imaging (MRI), and lengthens the time to a next relapse [2].

Statins are lipid-lowering oral drugs which inhibit the 3-hydroxy-3-methylglutaryl-coenzyme A (HMG-CoA-) reductase, the main regulatory enzyme of cholesterol biosynthesis. In addition to their lipid-lowering effects, statins have anti-inflammatory and immunomodulatory properties [3].

In experimental allergic encephalomyelitis (EAE), the animal model of MS, oral statins attenuate the severity of disease progression by preventing or reversing chronic or relapsing paralysis. Statin-treated animals show a delayed onset of first clinical signs and milder clinical signs [4-6].

In an open-label, single-arm study of oral simvastatin (80 mg/d) in patients with RRMS, the mean number and volume of lesions enhancing with gadolinium (Gd) compared to pre-treatment brain MRI scans declined by 44% and 41% respectively [7].

In a phase II open-label baseline-to-treatment trial of high dose atorvastatin monotherapy (80 mg/d) in RRMS there was a significant decrease in the number and volume of Gd-enhancing lesions in 24 RRMS patients, and a trend towards less Gd-enhancing lesions in 12 patients treated with the combination of atorvastatin (80 mg/d) and IFNB [8].

An open label study of INFB-1a and atorvastatin in 34 RRMS patients reported good tolerability and no significant difference in the clinical outcome measures between the patients who received IFNB-1a and atorvastatin vs. patients receiving IFNB-1a alone [9].

An interim analysis of the SIMCOMBIN study with 47 patients randomized to IFNB-1a or the combination of IFNB-1a and simvastatin 80 mg/d reported a similar annualized relapse rate [10]. Furthermore, two interim safety analyses of the current SWABIMS study regarding the primary endpoint showed no safety concerns.

On the other hand, in a double blind, randomized, placebo controlled study of twenty-six patients divided in three groups receiving IFNB-1a (44 ug thrice weekly) and 40 mg atorvastatin per day (7 patients), 80 mg atorvastatin per day (10 patients) or placebo (9 patients) for 6 months, the combination of atorvastatin (40 or 80 mg) and IFNB-1a resulted in increased MRI and clinical disease activity [11]. In addition, there are experimental studies, that suggest a negative impact of statins on oligodendrocytes and myelin formation with impaired remyelination [12,13].

Due to the differing results, bigger studies are needed to provide better evidence on the therapeutic potential of statins in MS. SWABIMS will give further information of the efficacy, safety and tolerability of atorvastatin 40 mg p.o. daily and IFNB-1b e.o.d compared to monotherapy with IFNB-1b e.o.d. in patients with RRMS.

## Methods/Design

**Figure 1:**
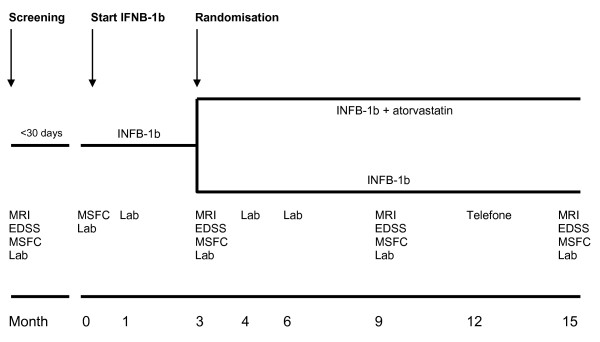
**SWABIMS study design**. Lab, laboratory tests; INFB-1b, Interferon beta-1b; MRI, magnetic resonance imaging, MSFC, multiple sclerosis functional composite; EDSS, Expanded Disability Status Scale

SWABIMS is a multi-centre, randomized, parallel-group, rater-blinded Phase IIb-study conducted in eight hospitals in Switzerland. Approximately 80 treatment naïve patients with RRMS (according to McDonald's criteria), respecting all inclusion and exclusion criteria, will receive IFNB-1b for 3 months. Afterwards, they will be randomized into two equal-sized parallel arms, receiving atorvastatin 40 mg/d or not in addition to IFNB-1b for another 12 months (Figure 1).

Patients and treating physicians are aware, whether atorvastatin is added to IFNB-1b or not. Placebo is not dispensed. Trained and certified examining physicians, responsible for MRI evaluation and disability scoring (Expanded Disability Status Scale [EDSS]; Multiple Sclerosis Functional Composite [MSFC]) are blinded to the treatment assignments (= rater-blinded).

The study is conducted in accordance with the ethical principles of the Declaration of Helsinki, the ICH-GCP Guidelines of 17 Jan. 1997 (G.U. n 191, 18 Ago 1997) and with the approval of the local ethics committees and SWISSmedic, the Swiss authorities for the authorisation and supervision of therapeutic products (Schweizerisches Heilmittelinstitut, Hallerstrasse 7, 3000 Bern 9, Switzerland; Reference number: 2005DR2119).

### Inclusion criteria

Patients with relapsing-remitting forms of MS according to McDonald's criteria with disease duration > 3 months, at least 1 relapse in the past two years, ≥ 3 lesions on spinal or brain-MRI or both, baseline-EDSS score from 0 to 3.5 (inclusive), age from 18 to 55 years and normal blood cell count, renal, and hepatic function values are enrolled.

Women of child-bearing potential have to agree to avoid pregnancy with appropriate means while on study, and prior to study entry a negative pregnancy test is mandatory.

The main **exclusion criteria **are primary or secondary progressive multiple sclerosis (PPMS, SPMS), clinically isolated syndrome (CIS), any disease other than multiple sclerosis that would better explain the patient's signs and symptoms, uncontrolled medical or psychiatric conditions, drug abuse, previous therapy at any time with monoclonal antibodies, mitoxantrone, cytotoxic or immunosuppressive therapy (excluding systemic steroids), total lymphoid irradiation as well as immunomodulatory therapy with IFNB, azathioprine or glatiramer acetate for 12 months before start of the study, if the previous treatment has been administrated for ≥12 months.

The **primary endpoint **is the disease activity measured by the proportion of patients with new T2 lesions after 15 months of treatment compared to baseline at month 3, when patients are randomized to receive atorvastatin or not.

**Secondary endpoints **are Gd-enhancing lesions on T1-weighted images, change of total T2 lesion volume (burden of disease, BOD), cortical atrophy (changes in brain volume, changes in grey matter and white matter), clinical disease progression (EDSS, MSFC), number of relapse-free patients, relapse rate, time to first relapse, and serial levels of neutralizing antibodies (NAbs).

The Multiple Sclerosis Functional Composite (MSFC) contains the "Timed 25-foot Walk", "9-Hole Peg Test" and the "Paced Auditory Serial Addition Test" (PASAT) and is, like the EDSS, performed according to international standards [14,15].

### Treatment

At screening visit, each patient has to provide written informed consent to the study and an unique patient-identification number is assigned. Afterwards, inclusion and exclusion criteria, demographic variables (age [years], gender, race), medical history (history of MS, general medical history and physical examinations), physical examination and vital signs (height [cm], weight [kg], systolic and diastolic blood pressure [mmHg], body temperature [°C] and pulse rate [bpm]), electrocardiogram (ECG) and laboratory tests are obtained by a study nurse and the treating physician. MRI, EDSS and MSFC scoring are performed by the evaluating physician. Laboratory tests included haematology values (RBC, WBC, platelets, differential count, haemoglobin, haematocrit, absolute lymphocyte count), serum lipids (triglycerides, total cholesterol, free cholesterol, HDL, LDL), serum chemistry (sodium, potassium, chloride, calcium, urea-N, ALAT, ASAT, γ-GT, alkaline phosphatase, total and direct bilirubin, creatinine, uric acid, glucose, total protein, albumin, urinalysis (protein, glucose) and creatine phosphokinase (total CPK, isoenzymes).

The randomization of each patient is performed centrally by the responsible Clinical Research Organisation (CRO) after the baseline visit. Randomization is conducted in 4-block size, according to the randomization list (atorvastatin "yes" or "no") generated with "RANCODE Professional 3.6". This sealed randomization list is stored in the CRO's archive. At the screening visit, a unique patient-identification number is assigned to each patient by the study investigator. A special "subject enrolment form", including the name of the study site and the unique patient-identification number is faxed to the CRO by the investigator of the study site. The CRO performs randomization according to the randomization list, by using a consecutive "randomization form" (atorvastatin "yes" or "no"), which is faxed back to the study site.

Screening and baseline visit are performed within 30 days.

At baseline visit, inclusion and exclusion criteria are controlled, MSFC scoring is repeated and MRI has to be accepted centrally by the responsible neuroradiologist. Afterwards, IFNB-1b is dispensed to all patients. Starting with 0.0625 mg (2 million units; 0.25 mL) e.o.d., IFNB-1b is weekly increased by 0.0625 mg to a final dose of 0.25 mg (8 million units; 1 mL).

At month 3, atorvastatin at a daily dose of 40 mg p.o. is dispensed to patients who were randomized to the atorvastatin-group, while IFNB-1b is planned to be on full dosage at that time point. The patients who do not receive atorvastatin continue monotherapy with full-dose IFNB-1b.

Regular visits at months 1, 3, 4, 6, 9, 12 and 15 are scheduled for the assessment and collection of EDSS, MSFC, MRI, NAbs, laboratory tests as well as other efficacy and safety data (Figure 1).

**Unscheduled visits **are performed in case of new, re-occurring or worsening neurological symptoms suspicious of relapses or medication side effects. Physical examination with vital signs, EDSS and laboratory tests are routinely performed.

### Relapse definition and treatment

All of the following criteria (1. to 3.) have to be met for a relapse to be ascertained: 1. neurological abnormality, either newly appearing or re-appearing, lasting for > 24 hours and being separated by at least 30 days from onset of a preceding clinical event; 2. Absence of fever or known infection (fever = > 37.5°C); 3. Objective neurological impairment, correlating with the patient's reported symptoms and increasing the total EDSS score and at least one of the functional systems of the EDSS score. Fatigue, mental symptoms, and/or vegetative symptoms without any additional symptoms are not classified as a relapse. Relapses are recorded and treated with intravenous (iv) methylprednisolone at a daily dose of 500 mg for five consecutive days followed by tapering-out with oral prednisolone.

All **MRI scans **are performed according to a standardized MRI protocol and will be assessed centrally for number and volume of lesions and for normalized brain volume at the Department of Neuroradiology of the University of Bern [16,17]. Each site has to submit a test scan for approval of scanning and data transfer prior to study start and all scanners should have at least 1.5 Tesla magnetic field strength. MRI scans of the brain will be performed at screening in order to check MRI inclusion criteria, and this scan will also serve as the first baseline scan. A second baseline scan, which will be used for assessment of endpoints, will be acquired 3 months later.

The following order for sequences has to be kept. 1. T1-weighted axial scout; 2. T1-weighted coronal scout; 3. T1-weighted sagittal scout; 4. Rapid T1-weighted SE or T2-weighted FSE/TSE axial (for repositioning check); 5. T2-weighted FSE/TSE; 6. T1-weighted SE post gadolinium. T1-weighted sequences of MRI scans are performed with concentrated (1 mol/L) gadobutrol at a dose of 0.2 mmol of gadolinium per kilogram of body weight.

Two experienced observers blinded to both clinical events and treatment are responsible for lesion identification and counting of all scans. Doubtful cases are assessed by consensual agreement. The observers are using a semi-automated program that identifies lesions of T2-weighted images. All lesions identified by the program have to be accepted by the investigators as such and investigators can add lesions not identified by the program. The program is also able to tell the examiner whether lesions on contiguous slices are part of one or several lesions and how many lesions have to be counted.

A single T2 lesion is defined as an area of increased signal on a given 3 mm axial image, which should be seen on both T2- and proton density-weighted images and which is not part of normally hyperintense structures. A single enhancing lesion is defined as an area of enhancement seen on a 3 mm axial image, which is referable neither to normally enhanced structures, nor to contrast migration within vessels. Lesions that change during the study are categorized as new, enlarging, or recurrent.

### Study management

The sponsor of this study is the Department of Neurology of the University Hospital in Bern, Switzerland, represented by the principal investigator Heinrich Mattle. The sponsor is responsible for the management of the entire study including data analysis and interpretation. The study is conducted independently of the two pharmaceutical companies which provide unrestricted grants for performing the study.

The clinical research organisation (CRO) PharmaPart GmbH (Bahnhofstrasse 20, Postfach 173, 8800 Thalwil, Switzerland) is contracted to help for data management, data analysis, randomization, visit tracking, drug distribution, case report forms (CRF), adverse and serious adverse event coding and others. The study statisticians will be blinded to the treatment-specific data throughout data collection process and analysis planning.

All laboratory analyses except NAbs are performed centrally by Viollier AG (Spalenring 145, Postfach, 4002 Basel, Switzerland). NAbs are assessed at the Laboratorio di Neurobiologia Clinica, Ospedale San Luigi Gonzaga Regione Gonzole, 10, 10043 Orbassano (TO), Italy. The cytopathic effect (CPE) assay is used to detect NAbs as recommended by the World Health Organization (WHO) [18]. Data from the neutralisation assay are reported as the reciprocal of the highest dilution of serum inducing 50% neutralisation (that is, neutralising 10 U/ml of IFN activity to an apparent 1 U/ml of activity). The neutralisation titre of a serum sample is calculated according to Kawade's formula and expressed in laboratory units (LU). A concentration of > 20 LU/ml is considered as a positive result. The IFNB used as reference or calibration standards in the CPE assay are the commercially available preparations of Betaferon, Avonex, and Rebif intended for clinical use [19].

### Statistical analysis

#### Determination of sample size

Data from completed clinical trials suggest that atorvastatin has the potential to increase the proportion of patients without any new MRI activity by 30% compared to the IFNB-1b alone. To show such an effect in a trial with 80% power and a significance level of 5% a sample of 76 patients is required. To allow a withdrawal rate of up to 5% during the trial, it is planned to enter approximately 80 patients.

All statistical analyses will be appropriate to the nature and distribution of the data collected. Categorical data will be described by frequency and percentage, continuous data by mean, standard deviation, minimum, 1^st ^quartile, median, 3^rd ^quartile and maximum. Hypothesis tests will be carried out with an α level of 0.05, two-sided. All inferential analyses will be presented by p-values, point estimations and two-sided 95% confidence intervals for the treatment differences. In case that the assumption of normality in the linear models is not fulfilled, transformations of the data or non-parametric approaches like the Wilcoxon signed rank test will be considered.

#### Analysis sets are defined as follows

All treated patients population (AT): All enrolled patients who receive any treatment and who have at least one follow-up efficacy observation independently whether they are randomized. Intention-to-treat population (ITT): All randomized patients, who have at least one follow-up efficacy observation. Per-protocol population (PP): The per-protocol set includes all patients who essentially complete the randomized part of the study in compliance with the protocol and who report no major violation.

The **primary endpoint **is the proportion of patients with new T2 lesions at month 15 compared to the T2 lesions at month 3 when patients are randomized to receive atorvastatin or not. Based on a logistic regression model with the factors treatment and gender and the covariates number of T2 lesions at baseline, number of Gd-enhancing lesions at baseline, relapse rate at baseline, baseline EDSS and time since MS diagnosis the two-sided hypothesis of equality between the two treatments are tested at an α-level of 0.05. The results are presented as odds ratios and the associated two-sided 95% confidence intervals and p-values. Furthermore, Fisher's exact test for proportions will be performed to test for the un-adjusted treatment effect.

Depending on the distribution, **secondary efficacy endpoints **are analyzed with analysis of covariance or logistic regression models or exact Fisher's exact tests. Time to relapse is analyzed with non-parametric methods for failure time data (Wilcoxon test) and illustrated by a Kaplan-Meier plot.

Missing data including missing data because of drop-outs on the primary endpoint (new T2 lesions at month 15) are replaced with MRI data from month 9. The same approach is used for the other efficacy endpoints. Missing values for other parameters will be treated as missing, except for severity and relationship of adverse events to study drugs. The number of missing and non-missing values will be given.

All safety and tolerability assessments are based on the ITT population and presented by treatment group. Adverse events are coded using the MedDRA 11.0 dictionary and concomitant medication using WHO-Drug 2007.1. Treatment emergent adverse events (randomized part of the study) are summarized by presenting the number and percentage of subjects having an event and the number and percentage of events in each system organ class and preferred term for each treatment group. Subjects who have multiple events in the same system organ class and preferred term are counted only once at each level of summation (overall, by system organ class, and by preferred term).

## Discussion

Statins have anti-inflammatory and immunomodulatory properties in addition to their lipid-lowering effects. The effects of statins on the course multiple sclerosis are still unknown. Experimental and clinical studies have given differing results and results from randomized trials are missing.

The Swiss Atorvastatin and Interferon Beta-1b Trial In Multiple Sclerosis (SWABIMS) is a multi-centre, randomized, parallel-group, rater-blinded Phase IIb-study conducted to assess the efficacy, safety and tolerability of atorvastatin 40 mg (p.o.) daily and IFNB-1b e.o.d compared to monotherapy with IFN-1b e.o.d. in patients with RRMS.

SWABIMS will likely give further information about the therapeutic potential of atorvastatin 40 mg p.o. daily as add-on therapy to IFNB-1b e.o.d in patients suffering from RRMS. In addition, SWABIMS will provide important safety and tolerability data of atorvastatin in patients with RRMS.

## Competing interests

Heinrich Mattle has received honoraria for lectures from Bayer-Schering, Biogen-Dompé, Merck-Serono, and Sanofi-Aventis and a research grant from Bayer-Schering and Pfizer to perform SWABIMS. Christian Kamm has no competing interests.

## Authors' contributions

All authors read and approved the final manuscript. Heinrich Mattle and Christian Kamm designed, organised, and managed the study. Heinrich Mattle is principal investigator.
